# Hybridization and extinction

**DOI:** 10.1111/eva.12367

**Published:** 2016-02-22

**Authors:** Marco Todesco, Mariana A. Pascual, Gregory L. Owens, Katherine L. Ostevik, Brook T. Moyers, Sariel Hübner, Sylvia M. Heredia, Min A. Hahn, Celine Caseys, Dan G. Bock, Loren H. Rieseberg

**Affiliations:** ^1^ Department of Botany and Biodiversity Research CentreUniversity of British ColumbiaVancouverBCCanada; ^2^ Department of Bioagricultural Sciences and Pest ManagementColorado State UniversityFt CollinsCOUSA; ^3^ Department of BiologyIndiana UniversityBloomingtonINUSA

**Keywords:** conservation, demographic swamping, gene flow, genetic swamping, hybrid fitness, introgression, invasive species, outbreeding depression

## Abstract

Hybridization may drive rare taxa to extinction through genetic swamping, where the rare form is replaced by hybrids, or by demographic swamping, where population growth rates are reduced due to the wasteful production of maladaptive hybrids. Conversely, hybridization may rescue the viability of small, inbred populations. Understanding the factors that contribute to destructive versus constructive outcomes of hybridization is key to managing conservation concerns. Here, we survey the literature for studies of hybridization and extinction to identify the ecological, evolutionary, and genetic factors that critically affect extinction risk through hybridization. We find that while extinction risk is highly situation dependent, genetic swamping is much more frequent than demographic swamping. In addition, human involvement is associated with increased risk and high reproductive isolation with reduced risk. Although climate change is predicted to increase the risk of hybridization‐induced extinction, we find little empirical support for this prediction. Similarly, theoretical and experimental studies imply that genetic rescue through hybridization may be equally or more probable than demographic swamping, but our literature survey failed to support this claim. We conclude that halting the introduction of hybridization‐prone exotics and restoring mature and diverse habitats that are resistant to hybrid establishment should be management priorities.

## Introduction

It has long been recognized that hybridization, defined here as mating between genetically distinguishable populations, can have a variety of evolutionary outcomes (Stebbins 1959; Abbott 1992; Arnold 1996). These include outcomes that maintain or increase diversity such as stable hybrid zones, the evolutionary rescue of small inbred populations, the origin and transfer of adaptations, the reinforcement of reproductive isolation, and the formation of new hybrid lineages (Anderson 1949; Ellstrand and Schierenbeck 2000; Mallet 2007; Abbott et al. 2013; Frankham 2015). Alternatively, hybridization can decrease diversity through the breakdown of reproductive barriers, the merger of previously distinctive evolutionary lineages, and the extinction of populations or species (Rieseberg et al. 1989; Ellstrand 1992; Levin et al. 1996; Rhymer and Simberloff 1996; Allendorf et al. 2001; Buerkle et al. 2003; Vuillaume et al. 2015). While our review focuses on extinction through hybridization, we consider its likelihood in the context of hybridization's many potential outcomes and the conditions that may favor one particular outcome over another.

**Figure 1:**
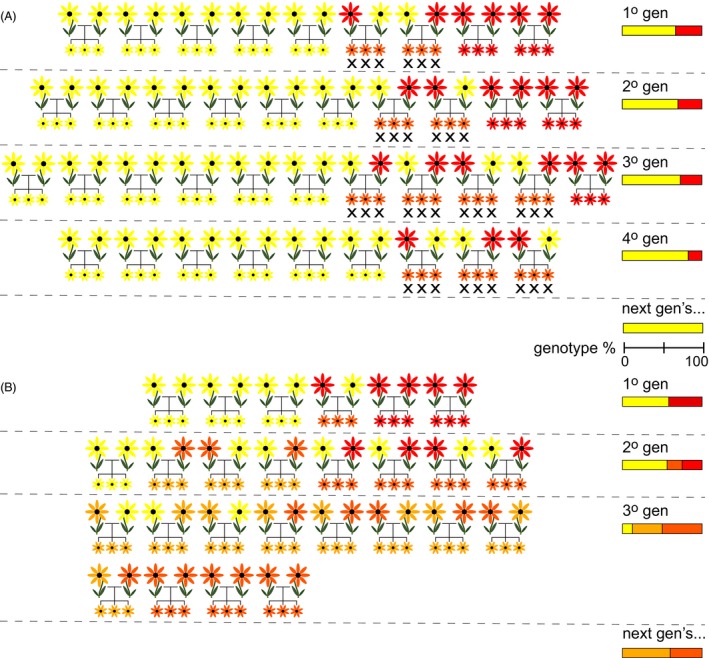
When rare (red flowers) and common (yellow flowers) lineages come into contact, hybridization may result in the local (or global) extinction of the rare lineage through (A) demographic swamping, in which unfit hybrid individuals (light and dark orange flowers) are entirely removed and with them all rare lineage alleles or (B) genetic swamping, in which hybrids are at least partially fertile and viable and replace pure parental genotypes. Note that demographic swamping results in population or lineage extinction, whereas genetic swamping results in the extinction of pure parental genotypes (i.e., genome extinction), but not of the alleles themselves. Rare, common, and hybrid genotype percentages per generation are represented in the color‐coded bars on the right side of both panels.

There are two main mechanisms by which hybridization can lead to extinction. If hybrid fitness is strongly reduced relative to that of parental individuals (i.e.*,* outbreeding depression), and hybridization is common, population growth rates of one or both parental lineages may decline below replacement rates due to wasted reproductive effort, leading to extinction (Fig. 1A). Following the terminology of Wolf et al. (2001), we refer to this mechanism as demographic swamping. On the other hand, if outbreeding depression is less severe, and population growth rates exceed replacement rates, then one (or both) of the parental lineages may be replaced by hybrids, a process typically referred to as genetic swamping (Fig. 1B). Admixture may occur even while parental phenotypic differences are maintained by divergent natural selection, potentially leading to the decoupling of genotype and phenotype. Because hybridization is an absorbing process, at some point all apparently phenotypically pure individuals of one or both parental lineages may have a hybrid ancestry, leading to the extinction of pure parental genomes (e.g.*,* Muhlfeld et al. 2014), but not necessarily parental alleles or traits.

Here, we review what we have learned about hybridization and extinction since the last comprehensive reviews of the topic 20 years ago (Levin et al. 1996; Rhymer and Simberloff 1996). At that time, only a handful of case studies had been carried out, and there were few theoretical studies of the process. Thus, these early reviews could provide little guidance on the likelihood of hybridization‐mediated extinction relative to other outcomes of hybridization, the relative importance of demographic versus genetic swamping, the roles of husbandry, invasive species, habitat disturbance, or climate change in the process, and so forth. To address these questions, we conducted a literature survey of studies on hybridization and extinction (below). After providing a brief overview of the results from this survey, we integrate our findings into a broader exploration of theory, as well as of the ecological, evolutionary, and genetic factors that may affect extinction risk through hybridization.

## Literature survey

Our survey was based on a Web of Science (Thomson Reuters) search for the keywords ‘hybridi*ation’ and ‘extinction’, including research articles published between January 1975 and May 2015. We determined that 357 papers were broadly relevant to this study based on information presented in the abstract. Of these, we excluded 66 reviews and 37 publications that focused on theoretical models. Each empirical publication was read by two people who independently scored the study for 17 categories (Table 1). If both readers considered a paper irrelevant to this review (e.g.*,* the hybridization under study was ancient or artificial), that paper was excluded (84 cases). Then, the two sets of categorizations were compared and corrected for consistency by three final ‘editors’ who collectively decided how to treat ambiguous cases. Finally, we combined multiple publications about the same species in the same location for a total of 143 independent cases (Table S1). We are aware that this survey is not comprehensive, as some relevant studies were not published in the Web of Science core collection or were not detected by our keyword search.

**Table 1:** Category definitions employed in the literature survey

Category	Definition and explanation of scoring procedure
Species	The species involved in hybridization. Each species was additionally scored for whether it was more widespread (globally common versus globally rare), more abundant in the area of study (locally common versus locally rare), a nonindigenous species (introduced), a widespread nonindigenous species (invasive), intentionally released into a habitat by humans (stocked), and at risk of extirpation or extinction due to genetic swamping and/or demographic swamping (threatened; see hybridization outcome category).
Hybridization outcome	Whether the predicted outcome of hybridization would be loss of genetically pure individuals for one of the species but preservation of the genetic material from that species in hybrid or introgressed individuals (genetic swamping), complete loss of the genetic material for one of the species (demographic swamping), preservation of genetically pure individuals for both species in the foreseeable future (no extinction threat), or a net fitness gain to one or both taxa without loss of taxonomic status (genetic rescue). In some cases, it was not possible to predict whether, upon extinction of genetically pure individuals, genetic material for the species would be preserved in hybrid individuals (genetic and demographic swamping).
Extinction level	For cases in which the likely outcome in the population of interest is extinction of one of the species, whether other populations of the same species not threatened by hybridization exist (local) or the threat includes all the known individuals for that species (global).
Taxa	Whether the hybridizing species are plants, invertebrates, or vertebrates.
Hybridization distance	Whether the hybridizing groups belong to the same (intraspecific) or different (interspecific) species.
Hybridization constancy	Whether hybridization has occurred over an extended period of time (continuous) or not (single event)**.**
Environment	Whether hybridization occurs within habitat typical to one or both of the hybridizing species (native), habitat with characteristics that fall between the two species’ typical habitats (intermediate), habitat that neither species typically occupies (novel), or more than one of these habitat types (multiple). In addition, whether the hybridization occurs in an area where the species are isolated and cannot have large population sizes (island).
Human involvement	Whether hybridization was caused or enhanced by human involvement. This includes where one of the hybridizing species is non‐native and its introduction was a consequence of human activities (species introduction), where hybridization was enhanced by habitat disturbance (habitat disturbance), and where one of the hybridizing species is actively managed by humans (husbandry; e.g.*,* crops, livestock, stocked fish, or game).
Release	Whether species introduction was intentional or unintentional.
Climate change	Whether hybridization was caused by or enhanced by global climate change or the effect of future climate change was explicitly examined.
Prezygotic barriers	Whether reproductive barriers that act prior to zygote formation such as ecogeographic, temporal, behavioral, and gametic isolation are present.
Postzygotic barriers	Whether reproductive barriers that act after zygote formation such as hybrid sterility, hybrid inviability, and hybrid breakdown are present.
Later generation hybrids	Whether the presence of individuals resulting from reproduction of F1 hybrids with other hybrids or individuals from parental species has been confirmed.
F1 asymmetry	Whether one of the hybridizing species was more likely to serve as the mother of hybrid progeny.
Backcross asymmetry	Whether biological mechanisms (i.e., independent from the abundance of the parental species) that result in preferential crossing of the F1 hybrids toward one of the parental species are present.
Cytoplasmic asymmetry	Whether there was increased representation of the cytoplasmic genomes of one of the parental species in hybrid progeny (including F1 hybrids and subsequent generations).
Nuclear asymmetry	Whether a higher proportion of hybrid individuals showed signs of introgression toward one of the parental species than expected.

**Figure 2:**
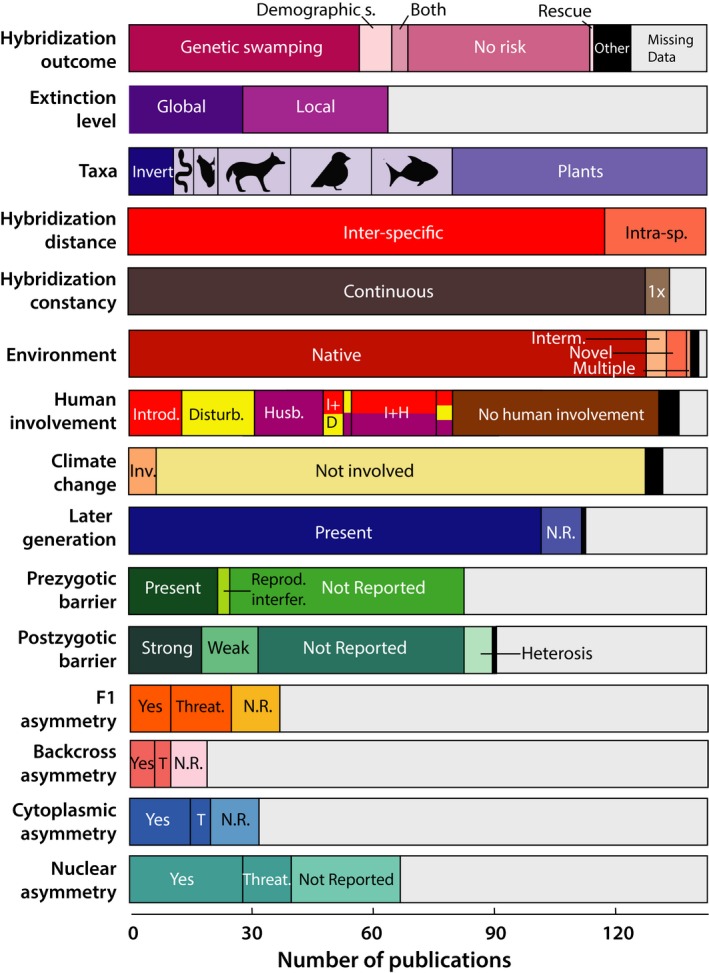
Overview of results from literature survey of 143 empirical papers (Table S1). Count data are shown for the different groupings that were scored for each category (Table 1). Black segments correspond to cases that did not fit well into defined groupings (other). Gray segments correspond to missing data, that is*,* cases in which the research article did not provide the information needed to classify the study. Articles that described the absence of a given phenomenon (e.g., asymmetric introgression) are noted as not reported (N.R.). For human involvement, groupings are as follows: species introduction (I, red), habitat disturbance (D, yellow), husbandry (H, purple), various combinations of these scenarios, or no human involvement. For asymmetry cases, introgression toward threatened species (Threat. or T) is noted. Further information on the species involved in hybridization and on the nature of species introductions can be found in Table S1.

The results from this survey (Fig. 2; Table S1) were challenging to evaluate statistically because of ascertainment bias deriving from our choice of search terms, missing information, subjectivity in assessment of hybridization outcomes, and correlations among categories. Nonetheless, we felt that the use of simple statistics—in this case Fisher's exact tests—was helpful in allowing us to distinguish between relatively stronger and weaker patterns in the data. Also, because we did not correct for multiple comparisons, false positives are likely. Indeed, if the most conservative correction for multiple tests were employed, none of our findings would be statistically significant.

### Overview of results

For studies where hybridization was considered to be an extinction threat (69 of 143 case studies), genetic swamping was the more common cause (87% of cases) and local (56%) rather than global extirpation was more frequently predicted (Table S1; Fig. 2). Genetic rescue appears to be an uncommon outcome of hybridization in the studies included in our survey (Fig. 2, and see below), but this might be due to ascertainment bias, since we included ‘extinction’ but not ‘rescue’ as a search term.

It is possible that our conclusions will hold for some taxa, but not others. In our survey, plant hybrids were most commonly studied, followed by fishes, birds, and mammals (Fig. 2). Extinction risk was more common in hybridizing vertebrates than plants (69% vs 52%), although not significantly so (*P *=* *0.11). This trend appears to be driven by fish (85%) and birds (79%). There were too few cases to make conclusions about invertebrates.

Despite our broadly inclusive definition of hybridization, most studies focused on the outcomes of interspecific (83%) rather than intraspecific hybridization (Fig. 2). However, the taxonomic status of the hybridizing species does not appear to affect predicted outcomes in our survey. In all but six cases, hybridization had occurred over an extended period of time (Fig. 2). This may increase the threat of outbreeding depression because foreign and possibly maladaptive alleles will be continuously introduced into the hybridizing populations. This conclusion is reinforced by the observation that hybridization mainly occurred in native (93% of studies) rather than novel environments, which presumably increases the likelihood that introgressed alleles will be maladaptive.

After excluding the five cases where the risk of extinction was unclear (‘other’), 72% of studies with human involvement reported an extinction threat while only 46% of studies without human involvement reported a threat (*P *=* *0.007). Among the cases where human involvement promoted hybridization, 55% involved husbandry or agriculture, 54% involved invasive species, and 36% involved habitat disturbance. Note that these factors are not independent; any one case could be affected by all three. Climate change, another consequence of human activities, was infrequently associated with hybridization in the current survey (Fig. 2).

Extinction risk through hybridization is lower when there are reproductive barriers (54%) and higher in the absence of reproductive barriers (67%), but this is not a significant difference (*P *=* *0.6). Nonetheless, this trend might partly account for the reduced extinction risk in plants versus vertebrates, since studies of the former are more likely to report the presence of one or more reproductive barriers (57% in plants versus 33% in vertebrates). Unfortunately, samples sizes were too limited to test the relative importance of prezygotic versus postzygotic barriers to extinction risk.

Most examples of extinction risk through genetic swamping report the presence of later generation hybrids (Fig. 2; Table S1). By contrast in all cases of demographic swamping, later generation hybrids are absent or there is reproductive interference.

All types of hybridization/introgression asymmetry are associated with extinction threat, but the details matter. In cases of F1 asymmetry, when the ‘common’ species tends to be the mother, there is little risk of extinction (14%). When the ‘rare’ species tends to be the mother, the risk is high (90%; *P *=* *0.004). This makes sense because females typically invest more resources into reproduction than males. Likewise, when nuclear introgression is into the ‘common’ species, there is less risk of extinction (8%) than when introgression is into the ‘rare’ species (67%) (*P *=* *0.019). This finding accords well with expectations from the process of extinction through genetic swamping.

## Theory

### Determinants of hybridization‐mediated extinction risk

While early reviews established hybridization as a threat to population persistence, questions remain about genetic and ecological factors that influence the likelihood and speed of extinction. Over the past 15 years, a number of theoretical studies have addressed these questions (Hall et al. 2006; Hall and Ayres 2008). We highlight below some of their predictions, support (or lack thereof) for these predictions from our literature survey (above), as well as important assumptions of the mathematical models used.

The first attempts at modeling hybridization–extinction dynamics had a genetic focus. Huxel (1999) considered hybridization between a locally common native and a locally rare invasive species that were fixed for different alleles at a single locus. Reproductive isolation was incorporated as F1 and backcross sterility. In addition, only invasive individuals were allowed to migrate into the environment where hybridization took place. With no hybridization and at moderate invader immigration rates and relative fitness, complete displacement of the native was always observed. Only when one or both of these parameters were considerably reduced did the native species persist. The risk of native extinction did not increase with hybridization when hybrids were sterile, as would be expected under demographic swamping. In Huxel's (1999) model, however, parental genotypes had large enough fecundities to maintain a constant population size. This is a fairly restrictive assumption that is unlikely to be met when native populations are already under some form of demographic pressure. When hybrids were fertile, extinction risk was increased when the fitness of heterozygotes was reduced (i.e.*,* underdominance). Conversely, simulations predicted that the native species can persist when heterozygote fitness is high, but this counterintuitive finding is an artifact of species membership being determined by a single bi‐allelic locus. In this situation, a large fraction of offspring from hybrid matings are considered to be pure parental genotypes, and the taxon that is initially more frequent will be less likely to go extinct.

This limitation was accounted for in subsequent genetic models. Ferdy and Austerlitz (2002) simulated hybridization in a community where the ranges of two partially interfertile plant species come into contact. Reproductive isolation was exclusively prezygotic and controlled by one or more unlinked bi‐allelic loci. Hybrids were classified according to the proportion of ancestry from the two parental species at these loci. Reproductive success between any two individuals was calculated based on a modeled interfertility parameter, as well as their ancestry: The probability of successful mating increased with the proportion of alleles that the two individuals shared at the reproductive isolation loci. Results emphasized the importance of the strength of reproductive barriers in preventing extinction, a prediction that is consistent with the results of our literature survey (above). Specifically, the two species were able to coexist when interbreeding was severely prevented. Conversely, high levels of interfertility invariably led to extinction. The genetic architecture of the isolating barrier also influenced the mode of species displacement. For the single‐locus architecture, the more common species replaced the rare one. More complex genetic architectures led to a gradient of intermediate phenotypes, and this gradient facilitated introgression. Replacement of both parental species by introgressed genotypes was the most frequent mode of extinction when considering genetic architectures of 2–8 loci, whereas extinction in scenarios with more loci always occurred by massive introgression (Ferdy and Austerlitz 2002).

Compared to genetic models, ecological models place a larger emphasis on life‐history traits. Wolf et al. (2001), for example, tracked the life cycle of a native and an invasive annual plant species that come into contact and hybridize. The two taxa varied in their relative abundances, were allowed different degrees of selfing, and were separated to various degrees by prezygotic (in the form of pollen competition) and postzygotic (in the form of hybrid fertility and competitive ability) reproductive barriers. In line with genetic models (e.g.*,* Huxel 1999; Epifanio and Philipp 2000; Ferdy and Austerlitz 2002), extinction risk of the native taxon was predicted to increase as its competitive ability and initial frequency decreased. Selfing rate also ranked high among parameters likely to prevent extinction, as expected given that selfing provides reproductive assurance in the face of declining population sizes.

Results from Wolf et al. (2001) also enabled predictions on the importance of reproductive barriers. For one, all else being equal, the speed of extinction was lowest when hybrids were sterile and increased with increasing hybrid fertility. Also, the model predicted that if reproductive barriers were asymmetric, hybridization leads to the extinction of the species that acts as the maternal parent, a prediction confirmed by our literature survey (above). Lastly, the model placed a higher premium on prezygotic than on postzygotic barriers for reducing risk of native extinction. This makes sense. When prezygotic barriers are missing, the native taxon is competing with—and can be extirpated by—both the invader and the hybrids. Ellstrand et al. (1999) and Fredrickson and Hedrick (2006) also emphasize the importance of prezygotic (especially premating) barriers in reducing extinction risk in the context of hybridization between crops and their wild relatives and between coyotes and red wolves, respectively.

Other predictions of Wolf et al.'s (2001) simulations were less intuitive. For example, under high levels of environmental stochasticity, the speed of extinction of the native taxon was predicted to increase. An important consideration when interpreting this result is that the default version of Wolf et al.'s (2001) model assumed equal initial frequencies of the hybridizing taxa. Also, after hybridization, the community consisted of three genotype classes, since all descendants of hybrid individuals were considered hybrids. In this case, following hybridization, native individuals are less numerous than non‐natives and therefore more prone to chance extinctions in unstable habitats. Likewise, the reverse outcome, that environmental stochasticity reduces the risk of native extinction, can be expected when the non‐native genotype is less frequent. Indeed, this was the predicted outcome in simulations by Hooftman et al. (2007), which considered hybridization between a common native taxon and a rare crop relative.

### Frequency of hybridization‐mediated extinction

Theorists have also investigated the frequency of hybridization‐mediated extinction relative to hybridization outcomes that maintain or increase biodiversity, although comparatively less effort has so far been devoted to this question. In Wolf et al.'s (2001) simulations, for example, extinction was invariably the outcome, unless two habitats were considered. If the hybridizing taxa were assigned to two different patches, and, concomitantly, if their competitive ability was at least three times higher in their local environment than in the adjacent one, a stable hybrid zone was formed. While in their study extinction was the dominant outcome, it is important to consider that Wolf et al.'s (2001) definition of species was conservative: any proportion of hybrid ancestry disqualified an individual from belonging to one of the two parental species.

A more inclusive species definition was used by Buerkle et al. (2003). In their simulations, species membership was decided based on genotypes at two fertility loci, analogous to two chromosomal inversions. Compared to previous models, Buerkle et al. (2003) also introduced one major variation, the possibility of homoploid hybrid speciation: Fertility was reduced in inversion heterozygotes, but could be restored to parental levels in novel homozygous genotypes. In addition, the two taxa differed at two habitat preference loci that conferred increased fitness in the local environment. The two species varied in relative abundances, with the common species occupying an area four times larger than that of the rare species. Finally, simulations were conducted with and without spatial separation of the two parental habitats.

Hybrid speciation was the least frequent outcome (2.1% of simulations), observed under conditions of no habitat separation but when F1 fertility and ecological selection were high, as well as with habitat separation when F1 fertility was high but when ecological selection was weak. The second outcome, extinction, was also infrequent (13.9% of simulations). It occurred only when F1 fertility was at its highest (90%), and there was no habitat separation. With habitat separation, extinction again occurred only at the highest F1 fertility level, although in this case moderate to strong ecological selection decreased the likelihood of extinction. Of the observed cases of extinction, all involved adaptive trait introgression: The common species acquired the locally adapted alleles from the rare species. Finally, Buerkle et al. (2003) estimated that by far the most common outcome of hybridization in their model, and therefore likely to be observed most often in nature, is the maintenance of a stable hybrid zone (84% of simulations). Indeed, the two hybridizing taxa were able to coexist across all levels of ecological selection and at all but the highest levels of interspecies fertility.

While the theory of extinction by hybridization has made important contributions to our conceptual understanding of this process, it has also made it clear that no single model can provide predictions that are universally applicable. This is because model assumptions, which are dependent on the biology of the hybridizing taxa, can have important bearing on the predicted outcome. The degree of spatial clustering is one such assumption. In the model of Wolf et al. (2001), for which native and non‐native individuals were randomly distributed, native taxon competitive ability was ranked highest among factors likely to prevent extinction risk. Buerkle et al. (2003), however, found hybrid fertility to be the most critical factor. As suggested by the authors, this difference can likely be traced back to the fact that, contrary to the simulations by Wolf et al. (2001), their model started with spatial clustering of the hybridizing taxa, and under spatial clustering fertility barriers are expected to create positive frequency dependence (Buerkle et al. 2003).

Keeping these limitations in mind, several more general predictions can be formulated based on results obtained from theoretical work. For one, given that the relative proportions of the interacting taxa in contact zones are consistently ranked high by modeling studies, extinction by hybridization is likely to be common in scenarios of species introduction characterized by heightened propagule pressure (although see Currat et al. 2008). While information on propagule size or number was not available in studies included in our literature survey, we did detect an association of hybridization‐mediated extinction risk with husbandry and biological invasions. In addition, this prediction has been validated in a number of well‐known examples, such as in mallard ducks and brook charr (see Husbandry section below). Extinction by genetic swamping should also be more common than extinction by demographic swamping, as replacement of the hybridizing parental taxa by hybrids is observed across a wider range of parameter values than is replacement by only one parental type. This prediction was confirmed by our literature survey. Note, however, that the definitions of ‘hybrid’ and ‘parental’ genotypes vary widely in models used to date. Also, prezygotic barriers to interspecific gene flow should have more weight in preventing risk of extinction than postzygotic barriers. Lastly, environmental changes are likely to have substantial effects on extinction risk through their effects on key factors in the models such as hybridization rates, relative fitnesses of hybrid and parental genotypes, and population growth rates (see *Habitat disturbance* section below).

## Human activities, hybridization, and extinction

Hybridization has long been known to be associated with human activities (Anderson and Stebbins 1954). Such activities may lead to contact between previously isolated taxa, making hybridization possible in the first place. In addition, human‐induced environmental changes may enhance the fitness of hybrids relative to that of parental genotypes and can generate new niches that are favorable to hybrid genotypes, thereby promoting the persistence of hybrid genotypes. Such changes are expected to increase extinction risk, and the association between human activities and extinction through hybridization was among the strongest found in our literature survey (see *Overview of results* section above). Below we describe some of the empirical literature that links human activities with extinction risk through hybridization, as well as some the factors underlying this linkage.

### Husbandry

The clearest link between human activities and extinction risk through hybridization come from actively managed species that are intentionally released into native habitats, where they may hybridize with native populations. Prominent cases include the stocking of fish populations for sport or commercial fishing, such as worldwide releases of salmonid species that hybridize with and thereby threaten native species. Examples include widely stocked rainbow trout (*Oncorhynchus mykiss*) and charr (*Salvelinus* sp.), which are eroding the genetic identities of native congeners (Rubidge et al. 2001; Allendorf et al. 2004; Rubidge and Taylor 2004; Sato et al. 2010). Similarly, intentional releases of game birds for hunting may pose a risk for wild relatives, such as black ducks (*Anas rubripes*) in America (Mank et al. 2004) and the endangered koloa (*Anas wyvilliana*) in Hawaii (Fowler et al. 2009), which are threatened by worldwide introductions of mallard ducks (*Anas platyrhynchos*) and subsequent hybridization. In addition, captive breeding programs such as that involving endemic Cuban crocodiles (*Crocodylus rhombifer*; Milián‐García et al. 2015), translocations of individuals for conservation purposes (Aitken and Whitlock 2013), and global species trade (e.g.*,* Perez et al. 2014) can result in potentially maladaptive hybridization with wild relatives.

Such intentional releases can lead to large differences in the abundance of native versus captive‐bred individuals. In mallard ducks, for example, the number of released individuals often exceeds that of wild individuals by factor of 10 (Čížková et al. 2012). Such high propagule pressure should increase the likelihood of hybridization—a prediction that has been validated in brook charr (*Salvelinus fontinalis*), where the number of stocking events in Canadian lakes was shown to be positively correlated with levels of hybridization (Marie et al. 2010). Husbandry may further increase the likelihood of hybridization by weakening the strength of reproductive barriers with close relatives (Rhymer and Simberloff 1996; Buerkle et al. 2003). For example, hybridization is sometimes used to improve the performance of captive‐bred stock, which may in turn facilitate hybridization with their parental species in the wild. This has been observed in partridges, where releases of chukar (*Alectoris chukar*) and red‐legged partridge hybrids (*Alectoris rufa*) appear to have facilitated hybridization with wild populations of the latter (Casas et al. 2012). Likewise, the breeding of cultivated plant species often includes one or more episodes of hybridization, which may weaken reproductive barriers. For instance, use of wild relative germplasm in the breeding of cultivated lantanas (*Lantana strigocamara*) appears to have predisposed commercial cultivars to hybridization and introgression with native congeners (Maschinski et al. 2010).

Hybridization involving captive‐bred individuals can have harmful consequences beyond the loss of genetic integrity (Rhymer and Simberloff 1996). In many cases, the stocked individuals differ genetically from the target population, which can result in outbreeding depression following hybridization (Muhlfeld et al. 2009). Selection pressures in captivity are likely to differ from those imposed by natural environments, which may give rise to genotypes that are maladapted to natural habitats (Piorno et al. 2015). Captive‐bred individuals may also suffer from low genetic diversity and inbreeding depression due to small population sizes and the use of relatively few individuals for breeding (Willoughby et al. 2015). Hybridization with maladapted or inbred individuals may lower the average fitness of populations, thereby threatening wild taxa. Our literature survey revealed that the intentional release of captive‐bred individuals was involved in 23% of cases (16/69) in which hybridization was considered to be an extinction threat. Changes in management practices, such as reduced stocking, more careful choice of stocked species and habitat, the release of nonreproductive individuals (e.g.*,* sterile triploids), could avert extirpation in many such cases (Thresher et al. 2013).

The unintentional releases of captive‐bred individuals appear to be less frequently associated with extinction risk through hybridization (17% of cases). Examples include escaped domesticated ferrets (*Mustela furo*) that hybridize with native European polecats (*M. putorius*) in Britain (Davison et al. 1999) and free‐ranging domestic cats that hybridize with European wild cats (*Felis silvestris silvestris*), threatening their genetic integrity (Oliveira et al. 2008). In plants, pollen and seed escapes from crops have become an agricultural concern, because most crops hybridize with wild relatives, potentially not only leading to the evolution of aggressive weeds, but also to the extinction of rare species (Ellstrand et al. 1999). Such escapes from domestication have been reported, for example, in cultivated carrots that hybridize with wild carrots in Denmark (Magnussen and Hauser 2007) or from plantations of *Eucalyptus nitens* into natural populations of the native congener *E. ovata* in Australia (Barbour et al. 2003). While many features of hybridization following intentional releases of captive‐bred individuals also apply to these unintentional escapes, the latter pose greater challenges for prevention and risk mitigation.

### Introduction of non‐native taxa

Of the 69 cases in our literature survey where hybridization was considered an extinction threat, 27 (39%) involved taxa that were not native to the region where hybridization occurred. Many of these include species that have been intentionally introduced and released, such as the aforementioned introductions of rainbow trout from its native range in the Pacific basin into lakes and rivers throughout the world (Fuller et al. 1999). Likewise, *Lantana strigocamara* is native to Central and South America, but was introduced to Europe in the 17th century, where it was bred and subsequently introduced as a cultivar to several continents (Maschinski et al. 2010). Other non‐native taxa have become introduced accidentally, such as the freshwater cyprinid *Pseudorasbora parva*, whose introduction accompanied the transplantation of carp species into new locations in Japan, where it hybridizes with an endangered congener *P. pumilla* (Konishi and Takata 2004). Similarly, discharges of contaminated shipping ballast led to the introduction of the European rockweed *Fucus serratus* to eastern North America, where it hybridizes with native *Fucus distichus* (Brawley et al. 2009).

Hybridization involving non‐native species may create unique problems. This is especially true for invasives, that is, taxa that have attained a widespread distribution in the introduced range. Because introduced taxa have not coevolved with native congeners, prezygotic reproductive barriers may be weaker, on average, than among native congeners. Also, invaders tend to be vigorous and abundant, spreading far beyond their initial point(s) of introduction. These factors can lead to high and potentially asymmetric hybridization rates (although see discussion of Currat et al. 2008 below), which may exacerbate extinction risk for rare natives. Lastly, non‐native species and their hybrids can serve as a bridge for gene flow between native species. For example, hybrids between the introduced white sucker (*Catostomus commersoni*) and the native flannelmouth sucker (*C. latipinnis*) in the Colorado River Basin have facilitated introgression between the latter and a previously isolated congener, *C. discobolus* (McDonald et al. 2008).

Well‐studied examples of invasive species threatening the genetic integrity of native taxa include hybridization and introgression between endemic Lesser Antillean iguanas (*Iguana delicatissima*) and invasive green iguanas (*I. iguana*), which have been introduced from French Guyana as stowaways on boats (Vuillaume et al. 2015). Similarly, hybridization with the invasive tiger salamander *Ambystoma tigrinum* in California threatens the declining native congener *A. californiense* (Riley et al. 2003). In plants, hybridization between invading Atlantic smooth cordgrass (*Spartina alterniflora*) and native California cordgrass (*S. foliosa*) threatens the latter with local extinction throughout the San Francisco Estuary (Ayres et al. 1999, 2004; Strong and Ayres 2013). It is noteworthy that both tiger salamander and California cordgrass are abundant species, illustrating that hybridization can be a threat to both rare and common species.

While the theoretical and empirical studies discussed above suggest that hybridization involving invasive species is frequently a threat to native congeners, this may not always be the case (Currat et al. 2008). Indeed, theoretical analyses of contact between native and invading populations suggest that introgression will mostly occur in the direction of the invading population (Currat et al. 2008). The reason for this is that native alleles that introgress into the invading population when it is at low density will be amplified by rapid growth of the invader, a phenomenon known as ‘allele surfing’. Thus, Currat et al. (2008) argue that the risk incurred by the native population when confronted with an invading taxon is primarily demographic rather than genetic. However, this conclusion requires interbreeding events to be frequent when the invading population is still at low density, an assumption that may be violated if the invader becomes abundant prior to contact or if reproductive barriers minimize interbreeding. Indeed, under arguably more biologically realistic assumptions, symmetric patterns of introgression are observed (Zhang 2014). While hybridization with an invasive species was associated with extinction risk in our literature survey (*P *=* *0.04), there were too few data to confirm (or refute) the predictions of Currat et al. (2008) regarding the direction of introgression.

Hybridization between invasive and native taxa is expected to become even more problematic in the future. Increased international trade and climate change may increase the number of invasive species and the likelihood of hybridization (Dukes and Mooney 1999), thereby exacerbating genetic risks for native taxa. In addition, some fraction of introduced but currently benign species may in the future become aggressive invaders (Sakai et al. 2001), possibly stimulated by hybridization itself (Abbott 1992; Ellstrand and Schierenbeck 2000). Management techniques and policies that halt the importation of exotic congeners will (obviously) also reduce the likelihood of hybridization with invaders. Strategies that maintain or restore mature and diverse communities can contribute as well, because they not only enhance community resistance to invasions (Shea and Chesson 2002; Rejmánek et al. 2005), but they also reduce the likelihood of hybrid establishment.

### Habitat disturbance

Anderson (1948) emphasized the importance of human disturbance as a driver of hybridization. Disturbed habitats are heterogeneous and ecologically unstable, and thus thought by Anderson to provide greater opportunity for hybrid establishment. However, the strength and appropriateness of the empirical data supporting this conjecture was recently questioned (Guo 2014). Our literature survey does confirm the predicted association between habitat disturbance, hybridization, and extinction risk, although the association appears weaker than for husbandry or the introduction of non‐native taxa (see *Overview of results* section above). Possibly this is due to inconsistent reporting of disturbance rather than a weak effect.

While the statistical link between human disturbance and hybridization was more tenuous than expected, numerous case studies report that hybrids are restricted to disturbed habitats. For example, *Kunzea sinclairii*, a rare shrub endemic to the rhyolitic rock outcrops on Great Barrier Island in northeastern New Zealand, is compatible with the more abundant close relative, *K*. *ericoides*, but hybrids are limited to disturbed sites created by fire or logging (de Lange and Norton 2004). Similarly, the endemic San Diego fairy shrimp *Branchinecta sandiegonensis* is threatened by hybridization from the widespread *B. lindahli* due to human activity disturbing the pristine vernal pools they live in (Simovich et al. 2013). In nondisturbed areas, hybrids of these species are not present, but in disturbed habitats a wide variety of hybrids and backcrosses are found.

Disturbance also can degrade parental habitat, thereby increasing the relative proportion of hybrids. This is seen in *Eucalyptus benthami*, which, due to habitat disturbance, is now restricted to the Australian Kedumba valley and three isolated stands. These isolated populations are vulnerable to heterospecific gene flow from *E. viminalis* due to density‐dependent effects, and indeed, smaller stands showed more evidence of introgression (Butcher et al. 2005).

Possibly the most compelling evidence linking habitat disturbance and hybridization derives from studies documenting the cessation of hybridization following habitat restoration. For example, Heiser (1979) reports on three sunflower (*Helianthus*) hybrid swarms that formed following habitat disturbance in the 1940s due to grazing, and/or trail and road construction. When he revisited the populations 22 years later, two of the sites had returned two predisturbance conditions and were dominated by plants that resembled one of the parental species. In contrast, one site remained disturbed and hybridization was still evident. Such observations also support a management strategy that includes habitat preservation and restoration. Mature, diverse, and undisturbed communities appear to be resistant to hybrid establishment and success. However, maintaining such communities will be challenging in the face of a growing human population, growing resource use, and climate change (below).

### Climate change

The threat of hybridization to species conservation may be amplified by climate change due to the breakdown of spatial, temporal, behavioral, or postzygotic reproductive barriers (reviewed by Chunco 2014). Spatial barriers can break down during range shifts as organisms track the changing climate (Anderson 1948; Rhymer and Simberloff 1996). For example, in Canada, southern flying squirrels (*Glaucomys volans*) have expanded their range north in response to climate change. This has brought them into contact with their congener, the northern flying squirrel (*G. sabrinus*) where hybridization has occurred (Garroway et al. 2010). Beyond bringing together previously allopatric species, range shifts can also increase the amount of range overlap between species, potentially increasing the hybridization rate beyond that at which selection can remove hybrids. This is seen convincingly in cutthroat trout (*Oncorhynchus clarkii*) of the Flathead River system (USA and Canada) (Muhlfeld et al. 2014). Rainbow trout (*Oncorhynchus mykiss*) were extensively stocked from the late 1800s to 1969, but by the 1980s only low (<2%) levels of hybridization with cutthroat trout were detected. As climate warming increased, so did the level of hybridization, and Muhlfed et al. showed that precipitation and summer stream temperature explained current introgression levels.

In many species, reproductive isolation is maintained by differences in the timing of breeding, which are known to be sensitive to climate change (Menzel et al. 2006; Cleland et al. 2007). These shifts in breeding are often idiosyncratic and thus can remove temporal barriers to gene flow (Parmesan 2007). Behavior‐mediated hybridization increases are predicted in spadefoot toads. Female Plains spadefoot toads (*Spea bombifrons*) prefer heterospecific Mexican spadefoot toads (*Spea multiplicata*) under low water conditions because development time is faster in hybrids and maturation time is limited by when the ponds dry out (Pfennig and Simovich 2002; Pfennig 2007). These conditions are likely to become more frequent under most climate change scenarios, thereby increasing hybridization (Seager et al. 2007; Chunco et al. 2012). This represents both a reduction in behavioral isolation and postzygotic isolation, as hybrids are selectively favored (or less disfavored) under climate change. This has also been seen in Daphnia, where an ice‐free winter caused a boom in hybrid genotypes (Zeis et al. 2010).

Although much effort has focused on how climate change can increase the effect of hybridization, it is also possible for climate change to reduce it. *Saxifraga hirsuta* is currently heavily introgressed by its congener *S. spathularis* in Ireland due to population density differences (Beatty et al. 2015). Climate projections and niche modeling suggest that *S. hirsuta*'s range will expand during climate change, potentially alleviating density‐dependent introgression from *S. spathularis*. Similarly, in damselfly, climate projections predict range contractions in two hybridizing taxa *Ischnura denticollis* and *I. gemina;* however, these range contractions reduce the potential range overlap between the species (Sánchez‐Guillén et al. 2014). Of course, although climate change may reduce hybridization in some cases, it may simultaneously threaten the same species in other ways.

In our literature survey, climate change‐induced hybridization is rare; only one paper had evidence that climate change caused or increased hybridization, although four others used climate predictions to speculate on increased hybridization in the future. There is still much work to be done to understand how severe a threat climate change‐associated hybridization is to threatened species. Future work could use climate predictions and niche modeling to estimate how frequently climate change will cause greater range overlap in threatened species (e.g.*,* Sánchez‐Guillén et al. 2014), as well as to develop strategies that maintain the resistance of ecological communities to hybrid establishment. The latter is challenging because the restoration of preexisting mature communities will not be possible. Rather, strategies must focus on keeping ecological communities mature, diverse, and healthy even while the identity and abundance of component species will be changing to match environmental conditions.

## Outbreeding depression and genetic rescue

While the focus of this review is on potential threats from hybridization, under certain conditions hybridization can ‘rescue’ (i.e., increase fitness of) small, inbred populations (Vilà et al. 2003; Madsen et al. 2004; Tallmon et al. 2004; Johnson et al. 2010). An open question is whether conservation managers have been too conservative in their use of hybridization for genetic rescue because of an exaggerated fear of outbreeding depression (Frankham 2015; but see Waller 2015).

### Outbreeding depression

Outbreeding depression refers to the decreased fitness of hybrids relative to their parents (Lynch 1991; Edmands and Timmerman 2003; Pekkala et al. 2012). Outbreeding depression can result from the disruption of local adaptation (Price and Waser 1979; Edmands 2007), the breakup of coadapted gene complexes (Templeton 1986; Lynch and Walsh 1998), and/or the expression of hybrid incompatibilities. The latter include Bateson–Dobzhansky–Muller (BDM) incompatibilities (Orr and Turelli 2001; Edmands 2007; Presgraves 2010), chromosomal rearrangements (White 1978; Rieseberg 2001; Fishman et al. 2013), and selfish genetic elements (Hurst and Schilthuizen 1998; Ågren 2013). If outbreeding depression is high, even low rates of hybridization may be fatal for small and isolated populations (Templeton 1986). As a consequence, risks from outbreeding depression are sometimes seen as on par with those posed by inbreeding depression (Templeton 1986; Edmands 2007).

Empirical estimates of outbreeding depression have focused mostly on early generation hybrids. This bias is partly for practical reasons, especially in long‐lived and difficult to propagate organisms, but also because outbreeding depression is expressed most strongly in early generations (Edmands et al. 2005; Frankham et al. 2011; Aitken and Whitlock 2013). For organisms that diverge rapidly in ecology or karyotype, such as annual plants (Lai et al. 2005; Fishman et al. 2013), outbreeding depression may be strongest in F1 hybrids due to maladaptation to parental habitats and/or underdominant chromosomal rearrangements. More commonly, fitness declines are greatest in F2 or F3 generations, where recessive BDM incompatibilities are exposed in homozygous genotypes and combinations of locally adapted alleles are broken up by recombination (Edmands 1999; Goldberg et al. 2005). Recovery of fitness usually has begun by the F4 generation and can be rapid (see discussion below).

Outbreeding depression typically increases with divergence of parental populations, at least for interspecific crosses (Moyle et al. 2004; Edmands et al. 2005). For crosses within species, some studies suggest that optimal fitness is reached at intermediate levels of divergence (Waser 1993; Trame et al. 1995; Hufford et al. 2012). This appears to be due to the expression of inbreeding depression in the closest crosses and heterosis at more intermediate genetic distances. However, other studies suggest that optimal fitness is reached at the lowest levels of divergence (Coyne and Orr 1997; Sasa et al. 1998; Edmands 1999), which is the expectation for crosses involving large, locally adapted populations that do not suffer from inbreeding depression.

These empirical results have been augmented by theory, which enable more general predictions and recommendations about outbreeding depression and its management. In an early study, Lynch (1991) developed a generalized model that accounts for the operation of both inbreeding and outbreeding depression. The model explores the evolution of autosomal loci with additive, dominance, and two‐locus epistatic variation, but it can be extended to other genetic architectures. If outbreeding depression results from the breakup of coadapted gene complexes, its expression in F1s is unlikely according to model predictions because the loss of favorable additive x additive epistasis would have to exceed twice the benefit obtained from beneficial dominance (i.e.*,* the masking of deleterious alleles). In the F2 generation, however, individuals have only half the heterozygosity of F1s, so a further reduction in fitness is expected unless there is highly favorable dominance x dominance epistasis.

These predictions are consistent with results from experimental crosses among copepod (*Tigriopus californicus*) populations along geographic and genetic divergence clines (Edmands 1999). F1 hybrids had increased mean fitness and reduced variance regardless of the level of divergence between parental populations. In contrast, fitness was reduced and variance increased in F2s, with stronger and more variable fitness breakdowns seen in progeny from more divergent parental populations. However, fitness was fully recovered in F3s, which implies that outbreeding depression may be limited in duration. Longer term experiments (up to 30 generations) largely confirmed this initial observation: Hybrid swarm replicates from one cross were equivalent to or exceeded the mid‐parent fitness, whereas replicates from another cross fell modestly above or below parental fitnesses (Pritchard and Edmands 2013). Similar results have been observed in long‐term experiments in plants, where outbreeding depression is typically purged after 5–10 generations of fertility or viability selection (reviewed in Rieseberg 1997).

Edmands and Timmerman (2003) used a computer simulation to explore these unexpected findings. In contrast to the empirical results, outbreeding depression persisted for many generations (375 on average). The magnitude of outbreeding depression increased with genetic divergence, the strength of local adaptation, and larger population size. Large population size also increased the duration of outbreeding depression (as did partial selfing), but duration was reduced if outbreeding depression was caused by the disruption of local adaptation (as opposed to the breakup of coadapted gene complexes) and if beneficial dominance was strong. Simulations also showed that very low rates of continuous gene flow could be as damaging as single episodes of extensive hybridization, since the former would continuously introduce maladaptive alleles into threatened populations. Hybridization had occurred over an extended period in all but a handful of the studies included in our literature survey, potentially amplifying the threat of outbreeding depression. On the other hand, our literature survey found little evidence that outbreeding depression was a frequent driver of extinction.

The apparent conflict between theory and empirical evidence regarding the expected duration of outbreeding depression appears to be resolved by Aitken and Whitlock (2013). Using a multilocus model in which outbreeding depression results from additive x additive epistasis, they show that if immigrants also carry locally beneficial alleles, hybridizing populations recover from outbreeding depression in fewer than 10 generations. This assumption seems reasonable given that adaptive introgression is commonly reported in the literature (e.g.*,* Whitney et al. 2006), and hybridization is frequently associated with environmental changes (Fig. 2). A complex genetic basis of outbreeding depression slowed recovery, but only modestly so when beneficial immigrant alleles were present. Bear in mind, however, that Aitken and Whitlock (2013) assumed a single bout of immigration, and recovery will be slowed by repeated episodes of hybridization. Nonetheless, it is clear that the fitness effects of hybridization are often surprisingly benign and that the duration of outbreeding depression can be unexpectedly short.

### Genetic rescue

We defined the ‘genetic rescue’ outcome in our literature survey as hybridization resulting in a net fitness gain to one or both taxa without threat of extinction. In classifying cases, we considered direct evidence for genetic rescue as well as more speculative reasoning by study authors. We note that this outcome could also be considered genetic swamping under our schema, as it is the contribution of alleles from one taxon to another that is responsible for increased fitness, for example*,* via relief of inbreeding depression. In genetic rescue, hybrids have equal or greater fitness relative to at least one of the parental lines, but this is not necessarily the case for genetic swamping. Also, for genetic rescue, (i) gene flow is limited over time or in degree; or (ii) selection is strong enough to maintain or re‐assemble the genomic and phenotypic differences between the parental lines.

Genetic rescue was a rare outcome in our literature survey: definitive in only one case, where hybridization was a human‐mediated, intraspecific, single event in a population suffering from inbreeding depression (Benson et al. 2011), fitting the more traditional, conservation management definition of genetic rescue (Whiteley et al. 2015), and putative in another two cases (Wachowiak and Prus‐Glowacki 2009; Harbicht et al. 2014). The rarity of this outcome in our survey may be the result of ascertainment bias resulting from our search terms (see above), but also may reflect the rarity of this outcome more generally in natural populations.

In a selective meta‐analysis, Frankham (2015) found increased fitness in 93% of cases where inbred populations were outcrossed. However, this dataset is restricted to cases screened for ‘low risk’ of outbreeding depression: wild, intraspecific, adapted to similar environments, having experienced gene flow within 500 years, and with no fixed chromosomal differences (Frankham et al. 2011; Frankham 2015). Our survey dataset consists primarily of interspecific hybridizers, many of which are adapted to different environments, relatively genetically divergent, or not experiencing inbreeding depression. It is perhaps not surprising that we observe so few cases of genetic rescue.

What is clear from both theoretical and empirical studies is that genetic rescue is highly dependent on the context of the hybridization (e.g.*,* Pickup et al. 2013) and the subsequent effects of selection (e.g.*,* Miller et al. 2012; Amador et al. 2014; Harbicht et al. 2014). Any outbreeding depression must be outweighed by the fitness benefits of hybridization, for example, by compensating for deleterious mutations that have accumulated in an inbred population (Frankham et al. 2011; Whiteley et al. 2015) or locally adaptive immigrant alleles (Aitken and Whitlock 2013). As a management strategy in conservation biology, hybridization is likely to be a useful tool in cases where populations are suffering from inbreeding depression, and the decision tree put forward by Frankham et al. (2011) for making such judgements appears to be effective. However, our literature survey suggests that genetic rescue is a less likely outcome of unintentional hybridization events, especially when hybridization is continuous or between highly divergent taxa.

## Conclusions

**Figure 3:**
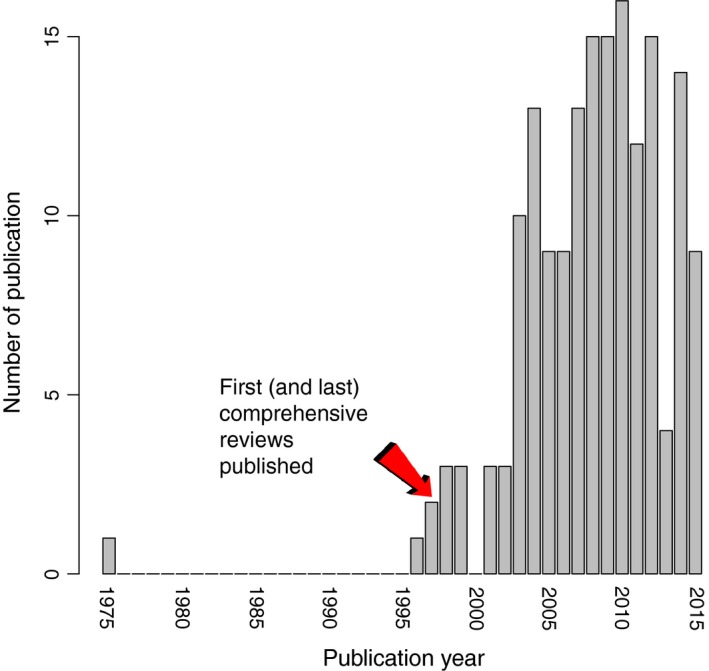
Number of relevant publications published between January 1975 and May 2015 that were detected by our Web of Science (Thomson Reuters) search for the keywords ‘hybridi*ation’ and ‘extinction’.

In 1996, two important reviews were published that established hybridization as an extinction threat (Levin et al. 1996; Rhymer and Simberloff 1996). These reviews appear to have stimulated research on the topic, with the majority of publications on hybridization and extinction appearing after 1996 (Fig. 3). Our review/analysis of this body of literature confirms a number of the predictions made by these earlier reviews, provides novel insights, sharpens management strategies, and highlights issues that require further study.

We specifically provide quantitative support for the long‐recognized association between human activities, hybridization, and extinction risk. Surprisingly, husbandry and species introductions contributed more to this association than did habitat disturbance, although this might be a consequence of ascertainment bias. We also confirmed predictions that extinction risk is higher in the absence of reproductive barriers and when hybridization/introgression is in the direction of the threatened species.

Our analysis/review further indicates that genetic swamping is more frequent than demographic swamping, extinction risk from hybridization is likely higher in hybridizing vertebrates than plants, the fitness consequences of hybridization can be surprisingly benign, and outbreeding depression will be short in duration if there is a single bout of immigration and immigrants carry locally adapted alleles.

Important questions remain. For example, it seems likely that climate change will amplify the threat of hybridization to species conservation, but support for this conjecture is currently weak. More generally, how do changes in the environment affect rates of hybridization and associated risks versus benefits? Theoretical and experimental studies imply that genetic rescue through hybridization may be equally or more probable than demographic swamping, but our literature survey failed to support this claim. At a more philosophical level, should we be opposed to extinction via genetic swamping if the hybrid entity that emerges is more variable and fit than one or both parental species? Such an entity might be better able to withstand future ecological and evolutionary challenges.

From a more practical standpoint, can our results inform management strategies? We contend that they can, in part by strengthening barriers to the introduction of hybridization‐prone exotics, sharpening decision making concerning the release of captive‐bred individuals and the appropriate use of hybridization for genetic rescue, and prioritizing restoration of mature and diverse habitats that are resistant to hybrid production and establishment.

Our review/analysis also points to gaps in our knowledge about the role/prevention of hybridization in species extinction and information and experiments that are needed to fill these gaps. Although there has been a large increase in the quantity of research on hybridization in species conservation since the mid‐1990s (Fig. 3), the quality of this work is highly variable. Many studies included in our literature survey provided little information about reproductive barriers and their strength, employed too few markers to allow robust inferences about patterns of introgression to be made (Figure S1), and failed to test the relative fitnesses of hybrid and parental genotypes in natural environments. Likewise, while numerous theoretical predictions have been made about the ecological, genetic, and evolutionary parameters that should affect hybridization‐associated extinction risk, experimental validation of these predictions is rare. Here, manipulative experiments involving model organisms would be helpful. Lastly, ecological studies are needed that manipulate natural communities to better understand what features of the communities make them more resistant to hybridization and to the establishment of hybrid genotypes.

## Data archiving statement

Data underlying results in this paper can be found in Table S1.

## Supporting information


**Figure S1** Number and kinds of markers employed in the studies of hybridization and extinction included in our literature survey. The number of publications is also shown.

**Table S1** Description of case studies employed in literature survey.
