# Supplementary material for: Hybridization and extinction
Source: Evol Appl. 2016 Feb 22;9(7):892–908. doi: 10.1111/eva.12367 (PMC4947151; doi:10.1111/eva.12367)
Supplement: Supplementary file 1 — Figure S1 Number and kinds of markers employed in the studies of hybridization and extinction included in our literature survey. The number of publications is also shown. [file EVA-9-892-s001.pdf]

Supplemental Figure S1

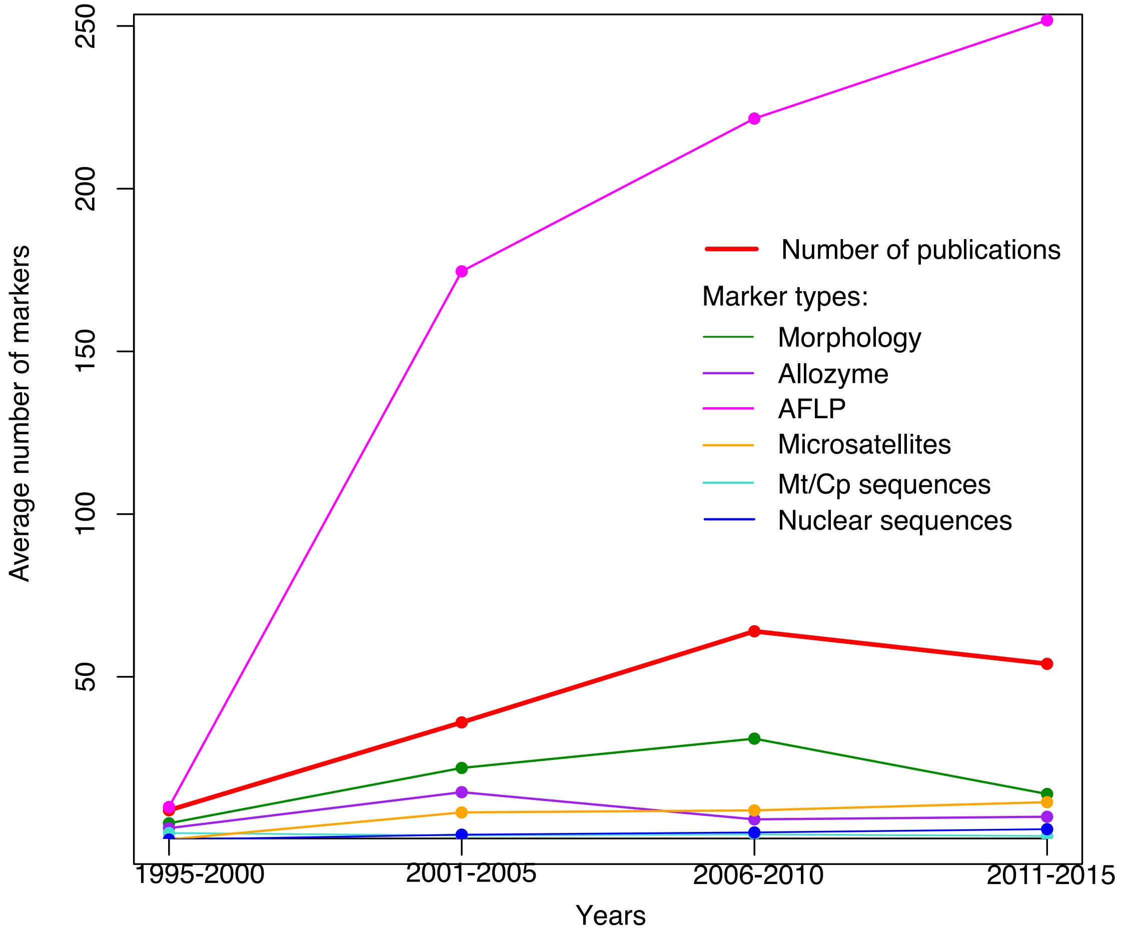

Figure S1 Number and kinds of markers employed in the studies of hybridization and extinction included in our literature survey. The number of publications is also shown.
