# Supplementary material for: Hybridization and extinction
Source: Evol Appl. 2016 Feb 22;9(7):892–908. doi: 10.1111/eva.12367 (PMC4947151; doi:10.1111/eva.12367)
Supplement: Supplementary file 2 — Table S1 Description of case studies employed in literature survey. [file EVA-9-892-s002.docx]

Description of case studies employed in literature survey.
